# An analysis of expressed sequence tags of developing castor endosperm using a full-length cDNA library

**DOI:** 10.1186/1471-2229-7-42

**Published:** 2007-07-31

**Authors:** Chaofu Lu, James G Wallis, John Browse

**Affiliations:** 1 Institute of Biological Chemistry, Washington State University, Pullman, WA 99164-6340, USA; 2 Department of Plant Sciences and Plant Pathology, Montana State University, Bozeman, MT 59717-3150, USA

## Abstract

**Background:**

Castor seeds are a major source for ricinoleate, an important industrial raw material. Genomics studies of castor plant will provide critical information for understanding seed metabolism, for effectively engineering ricinoleate production in transgenic oilseeds, or for genetically improving castor plants by eliminating toxic and allergic proteins in seeds.

**Results:**

Full-length cDNAs are useful resources in annotating genes and in providing functional analysis of genes and their products. We constructed a full-length cDNA library from developing castor endosperm, and obtained 4,720 ESTs from 5'-ends of the cDNA clones representing 1,908 unique sequences. The most abundant transcripts are genes encoding storage proteins, ricin, agglutinin and oleosins. Several other sequences are also very numerous, including two acidic triacylglycerol lipases, and the oleate hydroxylase (*FAH12*) gene that is responsible for ricinoleate biosynthesis. The role(s) of the lipases in developing castor seeds are not clear, and co-expressing of a lipase and the FAH12 did not result in significant changes in hydroxy fatty acid accumulation in transgenic Arabidopsis seeds. Only one oleate desaturase (*FAD2*) gene was identified in our cDNA sequences. Sequence and functional analyses of the castor *FAD2 *were carried out since it had not been characterized previously. Overexpression of castor FAD2 in a FAH12-expressing *Arabidopsis *line resulted in decreased accumulation of hydroxy fatty acids in transgenic seeds.

**Conclusion:**

Our results suggest that transcriptional regulation of *FAD2 *and *FAH12 *genes maybe one of the mechanisms that contribute to a high level of ricinoleate accumulation in castor endosperm. The full-length cDNA library will be used to search for additional genes that affect ricinoleate accumulation in seed oils. Our EST sequences will also be useful to annotate the castor genome, which whole sequence is being generated by shotgun sequencing at the Institute for Genome Research (TIGR).

## Background

The hydroxy fatty acid ricinoleate (12-hydroxy-octadeca-*cis*-9-enoic acid: 18:1-OH) is an important natural raw material with great value as a petrochemical replacement in a variety of industrial processes. Its derivatives are found in products such as lubricants, nylon, dyes, soaps, inks, adhesives, and biodiesel [1]. The seeds of castor plant (*Ricinus communis *L.) are the major source of ricinoleate, which constitutes about 90% of the total fatty acids of the seed oil. However, oilseed castor cultivation is limited to tropical and sub-tropical regions, and seeds are laboriously harvested by methods that are difficult to adapt to large-scale production. In addition, castor seeds contain the poisonous ricin as well as strongly allergenic 2S albumins, which pose health threats for workers during planting, harvesting and processing. It is therefore highly desirable to produce ricinoleate in temperate oilseed crops through genetic engineering.

Ricinoleate biosynthesis in castor seeds is catalyzed by an oleate Δ12-hydroxylase (FAH12), a close homologue of the oleate Δ12-desaturase (FAD2) [2]. The FAH12 adds a hydroxy group (-OH) to the twelfth carbon of oleic acid moieties esterified to the *sn*-2 position of phosphatidylcholine [3]. Expression of FAH12 in transgenic tobacco and *Arabidopsis *caused the accumulation of hydroxy fatty acids, but only to about 17% of total seed oil, far less than that in the native castor seeds [4-6]. To increase ricinoleate in transgenic oilseeds and create a castor oil replacement, it is necessary to better understand the mechanisms of lipid metabolism in castor seed. We are specifically interested in the expression profile of genes that are co-expressed with the *FAH12 *gene because some of these gene products may also contribute to ricinoleate accumulation in developing castor seeds. Expressed sequence tag (EST) analysis provides a convenient and efficient gateway for identification of genes expressed in specific tissues and cells as well as allowing characterization of the level of transcript expression [7]. Despite the availability of a small number (744) of ESTs from developing castor endosperm [8], and a more wealthy EST collection from leaves recently released by the Institute of Genome Research [9], gene expression information in developing castor endosperm is limited. There was no full-length cDNA resource in castor either. In this report, we sequenced the 5'ends of about 5,000 cDNA clones from a full-length cDNA library derived from developing castor endosperm, the storage organ in castor seed. We analyzed the abundance of specific cDNAs from 4,720 EST sequences. We found that the castor oleate desaturase (*RcFAD2*) sequence is much less abundant than that of the *FAH12 *in our cDNA sequences, suggesting a transcriptional control of these two genes in castor endosperm to favor ricinoleate accumulation.

## Results and discussion

### Single-pass sequencing of a castor full-length cDNA library

**Figure 1:**
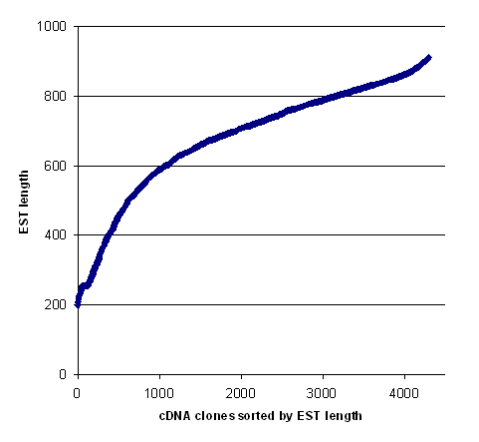
Distribution of sequence length of ESTs containing more than 200 nucleotides.

**Figure 2:**
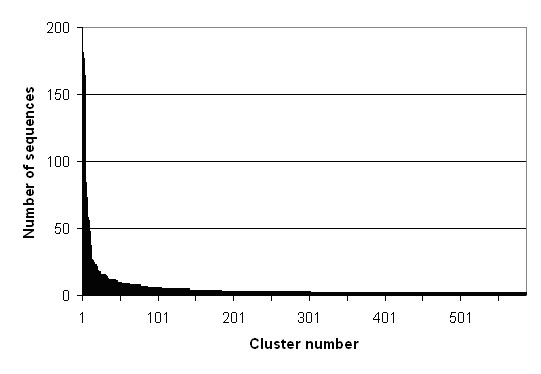
Distribution of EST clusters of more than 2 sequences.

In order to systematically analyze genes expressed in developing castor seeds and to facilitate functional analysis of the cDNA clones, we constructed an oriented full-length cDNA library in a lambda vector that incorporated the Gateway cloning system. The quality of this library was assessed by PCR and sequencing of the inserted cDNA clones. The length of insert cDNA clones ranged from ~600 bp to over 6 kb, which reflected the size distribution of the first-strand cDNA population. Moreover, many genes known to be involved in lipid metabolism are present in the library [6]. Our analysis after sequencing of 140 clones indicated that over 90% of the clones contain full-length protein coding sequences [6]. These observations suggested that there was not significant bias towards short cDNA clones during the full-length library construction. In this study, we sequenced the 5'-ends of about 5,000 plasmid clones that were excised from the amplified lambda library by the Gateway cloning process. To maximize the efficiency of cDNA sequencing, we used a sequencing primer located immediately adjacent to the 5'ends of cDNA inserts. This yielded 4,720 high quality (Phred Q>20 [10]) sequences, which included approximately 2.25 M castor sequence. Further examination resulted in 4,288 sequences that contained over 200 nucleotides with an average length of 679 nucleotides per EST (Fig. 1). Visual examination of 100 random sequences and their translated results using the translation tool  indicated that the average length of the 5'-untranslated region (UTR) is about 75 nucleotides. Cluster analysis and assembly of these sequences resulted in a total of 1,908 unique EST sequences with 587 contigs (30.8%) (Fig. 2) and 1,321 singletons (69.2%). We have deposited 4,288 sequences in the dbEST division of GenBank.

### Highly expressed genes mostly encode storage proteins and oleosins

The purpose of this study is to obtain a brief snapshot of genes expressed in developing castor endosperm, and to identify genes that may contribute to ricinoleate accumulation. We compared each unique EST sequence with the non-redundant (nr) protein databases of the NCBI and Arabidopsis proteins at TAIR using the BLASTX program. The results [see Additional file 1] indicated that about 95% of the sequences identified homologues in *Arabidopsis *or other organisms. The remaining 5% of the genes encode proteins that may be unique to castor, or to the *Euphorbiaceae*, since no homologues were found in the available databases. About 13% of the genes encode proteins whose functions in *Arabidopsis *or other organisms remain unknown. Table 1 lists the most abundant sequences (>10 EST counts) from the library. Similar to the ESTs in developing *Arabidopsis *seeds [11], genes encoding storage proteins are the most abundant ones in developing castor seed, comprising about 18% of the total. These proteins include *Ricinus communis *seed storage proteins, a legume-like protein and its precursor, and the allergenic 2S albumin and its precursor. Genes encoding the toxic proteins ricin and agglutinin are also highly expressed in developing castor endosperm (1.5% and 1.2% of total, respectively). This information is useful for the transgenic strategy to eliminate the toxic ricin and agglutinin and the allergenic 2S albumin from castor seeds [12]. On the other hand, normalization of the library by eliminating these highly abundant sequences before further sequence analysis will increase the efficiency of gene discovery, since genes expressed in fewer copies will be more readily detected.

**Table 1:** The most abundant sequences from a full-length cDNA library of developing castor endosperm

Cluster ID	No of ESTs	Arabidopsis homolog	Functional description of gene product
cn56	296	At5g44120	legumin precursor
cn69	193	At5g54740	2S albumin
cn55	164	At5g44120	seed storage protein [*Ricinus communis*]
cn67	170	At4g25140	Oleosin
cn22	106	At4g27140	2S albumin precursor (Allergen Ric c 1)
cn162	73	-	Agglutinin precursor (RCA)
cn161	56	At5g59680	Ricin precursor
cn18	48	At3g09390	Metallothionein-like protein
cn62	37	At4g27140	2S albumin
cn16	34	At3g01570	16.9 kDa oleosin
cn29	27	At4g27150	2S albumin precursor (Allergen Ric c 1)
cn123	26	At1g72330	alanine aminotransferase
cn167	25	At5g39850	40S ribosomal protein S9 (RPS9C)
cn209	25	At3g18280	Probable nonspecific lipid-transfer protein AKCS9 precursor (LTP)
cn82	23	At3g14360	lipase (class 3) family
cn267	23	At1g08360	60S ribosomal protein L10A (RPL10aA)
cn200	20	At1g13440	glyceraldehyde-3-phosphate dehydrogenase
cn137	19	At5g54770	Thiazole biosynthetic enzyme, chloroplast precursor
cn332	18	At2g36530	Enolase (2-phosphoglycerate dehydratase)
cn76	18	At1g65090	unknown protein
cn13	18	-	No hits
cn59	16	At1g62710	Vacuolar processing enzyme precursor (VPE)
cn120	16	At2g05920	subtilisin-like serine protease, putative
cn196	16	At3g02470	S-adenosylmethionine decarboxylase
cn115	16	At2g05990	enoyl-ACP reductase
cn93	16	At3g12120	oleate 12-hydroxylase – castor bean
cn91	15	At5g60390	elongation factor – alpha (EF-1-ALPHA)
cn201	15	At2g32060	putative 40S ribosomal protein S12
cn12	14	At1g54580	Acyl carrier protein 1, chloroplast precursor (ACP 1)
cn112	13	At1g43800	acyl- [acyl-carrier-protein] desaturase (stearoyl-ACP desaturase)
cn155	12	At1g77510	Protein disulfide isomerase precursor (PDI)
cn203	12	At3g55440	Triosephosphate isomerase, cytosolic (TIM)
cn402	12	At3g05590	60S ribosomal protein L18 (RPL18B)
cn113	12	At2g30200	malonyl-CoA:Acyl carrier protein transacylase
cn21	12	-	No hits
cn127	12	At5g13490	ADP, ATP carrier protein 1, mitochondrial precursor
cn142	12	At1g79550	cytosolic phosphoglycerate kinase 1
cn335	12	At2g36640	embryonic protein BP8
cn158	12	At1g43170	L3 Ribosomal protein
cn77	11	At5g63660	proteinase inhibitor se60-like protein
cn422	11	At1g67360	stress related protein -related
cn53	11	At5g39850	40S ribosomal protein S9 (RPS9C)
cn202	10	At5g12380	Annexin-like protein RJ4
cn192	10	At1g04820	alpha-tubulin
cn320	10	At4g11600	glutathione peroxidase, putative
cn324	10	At3g07565	OSJNBa0067K08.3 [*Oryza sativa *(japonica cultivar-group)]
cn105	10	At3g16640	Translationally controlled tumor protein homolog (TCTP)

Oil-body oleosin genes are also highly expressed, making up about 4% of the total sequences. The 209 ESTs for oleosins in the sequenced clones represent 6 different genes according to sequence similarity to *Arabidopsis *oleosin homologues. These genes are expressed at different levels. The castor oleosin *RcOLE2 *(accession No. AAR15172), a homologue of the Arabidopsis *At4g25140*, is the most abundant one (170 ESTs). There are 34 ESTs representing the *RcOLE1 *(accession No. AAR15171), a homologue of *At3g01570*. Others are much less abundant. Only two ESTs are homologous to *At5g51210*, and one EST each for the oleosins that are homologous to *At2g25890*, *At3g18570*, and *At3g27660*, respectively. In contrast, expression levels of different oleosins in developing *Arabidopsis *seeds vary less dramatically. For example, the EST counts for *At4g25140*, *At5g40420 *and *At3g27660 *are 9, 38 and 49, respectively from 10,522 sequences [11]. The relatively high abundant 21-KD oleosin gene (*At5g40420*) in *Arabidopsis *seeds is absent in our cDNA sequences of castor. These findings suggest that different oleosins may play different roles in oil accumulation in castor and *Arabidopsis *seeds. In our high-throughput screening experiment, we found that co-expressing RcOLE2 (an At4g25140 homologue) with FAH12 resulted in moderately increased hydroxy fatty acid accumulation in transgenic *Arabidopsis *seeds [6]. At4g25140 plays an important role in regulating oil body size in *Arabidopsis *seed [13]. The abundance of *RcOLE2 *in our EST collection suggests it may play a similar role in castor seed.

### The acidic lipases are highly expressed in developing castor endosperm

Besides storage proteins, oleosins, ricin and a metallothionein-like protein as listed in Table 1, there are several genes that are somewhat abundant in our cDNA library. These include lipid transfer proteins, genes encoding components of the protein biosynthetic apparatus such as alanine aminotransferase, ribosomal proteins, and elongation factor 1-alapha, as well as proteins involved in carbohydrate metabolism such as glyceraldehyde-3-phosphate dehydrogenase, enolase, and triosephosphate isomerase. The genes in this class also include the oleate hydroxylase (*FAH12*) and other genes of lipid metabolism such as acyl carrier protein (ACP), stearoyl-ACP desaturase, and malonyl-CoA:ACP transacylase.

Interestingly, as listed in Table 1, we identified a class-3 triacylglycerol lipase (cn82) that is highly abundant (23 ESTs) in our cDNA library. This gene, we termed *RcTGL3*, was recently characterized as an acidic triacylglycerol (TAG) lipase of the castor bean [14]. A close homologue of this gene (*RcTGL3-2*) with 87% sequence identity was also identified (cn81), and its full-length sequence was determined (GenBank accession No. EF071862). The *RcTGL3-2 *gene is moderately abundant in our cDNA library (8 ESTs). The more abundant *RcTGL3 *gene is specifically expressed in developing castor endosperm as revealed by RT-PCR analysis (data not shown; also see [14]). The function of a TAG lipase is to hydrolyze TAG into fatty acids and the intermediate products diacylglycerol or monoacylglycerol. The high level of expression of the TAG lipases along with many lipid synthetic genes in developing endosperm of castor seeds raised questions about their roles in seed development or lipid accumulation. Speculating that they might play a role in ricinoleate accumulation in castor endosperm, we transformed the two lipase homologues independently into a FAH12-expressing *Arabidopsis *line, CL37 [6], and the fatty acid methyl esters of the transgenic seeds were analyzed by GC. The fatty acid compositions of the transgenic seeds that co-expressed FAH12 and either lipase genes showed no significant difference from those of CL37 (data not shown). This result suggested that the lipases might not have significant contribution to fatty acid synthesis in transgenic Arabidopsis seeds. We did not pursue further studies of the transgenic lines since they had no effect on hydroxy fatty acid accumulation. Whether the transgenic lipase genes have altered lipase activities and their consequences on seed metabolism and physiology remain subjects of future investigations.

It is not clear why lipases express at such a high level of expression in developing seeds while lipid synthesis is actively taking place. The acidic lipase protein has also been detected in dry and germinating castor seeds [14], suggesting a role in breakdown of storage lipids to support post-germinative seedling development. However, the presence of a neutral or alkaline TAG lipase in castor seed and its predominant role in lipolysis [15] conflicts with this simple interpretation. Reverse-genetic analysis by knockout or knock-down of these genes in castor plant may provide an answer to the function(s) of the acidic lipases in developing seeds, as transformation technology has recently been extended to castor [16].

### The *FAD2 *gene is not highly expressed in developing castor seed

One of our purposes in analyzing ESTs was to identify genes that are important to lipid metabolism in castor endosperm. In contrast to a very high abundance of oleosins, and the moderately high abundance of some genes including the *FAH12 *and others that are listed in Table 1, most genes involved in lipid metabolism occur once or a few times in our EST data. Although about 3% of the genes we identified encode proteins involved in various aspects of lipid metabolism, they represent a small proportion of the approximately 150 lipid metabolism genes expressed in *Arabidopsis *seeds [17]. For example, genes encoding enzymes such as diacylglycerol acyltransferase and others known to play major roles in TAG biosynthesis were not detected by our EST analysis, although some were detected by PCR analysis of our library [6].

**Figure 3:**
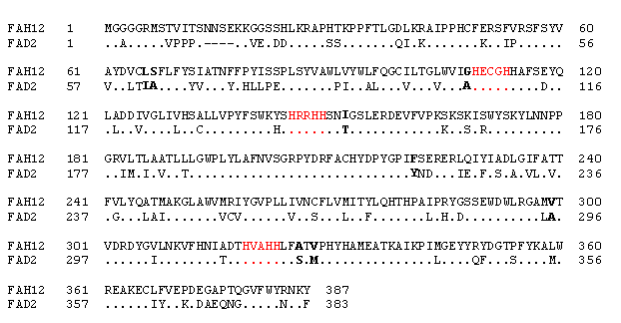
**Sequence comparison between the oleate hydroxylase (FAH12) and the oleate desaturase (FAD2) in castor**. The FAD2 is four amino acids shorter than the FAH12 at the N-terminus (shown by dashes). Identical amino acids are indicated by dots. The three regions containing histidine residues conserved among fatty acid desaturases are shown in red letters. The 8 amino acids in bold faces have been shown to be involved in determining the catalytic outcome of the desaturation/hydroxylation reactions [31].

**Figure 4:**
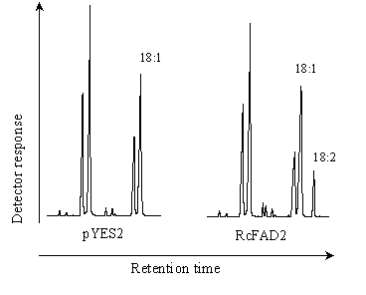
**Functional analysis of the castor FAD2 enzyme by heterologous expression in yeast**. Fatty acid methyl esters of yeast cells transformed with empty vector pYES2 (left) and *RcFAD2 *gene were analyzed by gas chromatography.

We identified only one cDNA clone amongst our ESTs encoding the yet uncharacterized castor FAD2 oleate desaturase, and determined the full-length sequence of this gene (GenBank accession No. EF071863). The deduced amino acid sequence of castor FAD2 shares a high level (74%) of identity to that of the FAH12 (Fig. 3). To confirm the functional identity of the castor FAD2 cDNA, we have cloned the corresponding ORF into the expression vector pYES2 (Invitrogen, CA) behind the inducible promoter GAL1, and transformed into *S*. *cerevisiae *cells. Yeast cells have been used successfully for functional expression of several plant microsomal desaturases including FAD2, as they act as a very convenient host due to its simple fatty acid profile, the presence of only one major fatty acyl desaturase, and the appropriate redox chain in a suitable membrane [18]. The fatty acid analysis of the transformant yeast cells grown in galactose-containing medium showed the presence of a new fatty acid, which was not present either in the wild-type yeast or in the control cells transformed with the empty vector pYES2. The new fatty acid was identified as linoleic acid (18:2) by GC-MS (Fig. 4).

**Figure 5:**
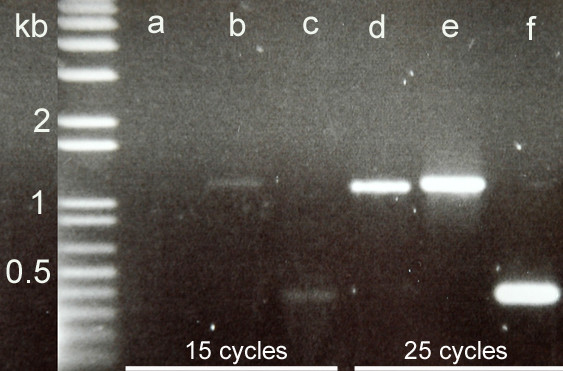
**Comparison of expression levels of castor *FAD2*, *FAH12 *and oleosin (*OLE2*) genes in developing endosperm by RT-PCR analysis**. (a, d) *FAD2*; (b, e) *FAH12*; (c, f) *OLE2*. PCR conditions are 94°C 30s, 55°C 30s and 72°C 1min for 15 cycles (a, b, c) or 25 cycles (d, e, f). Equal amount (3 μL) of PCR reactions (total 20 μL) were loaded for electrophoresis.

**Figure 6:**
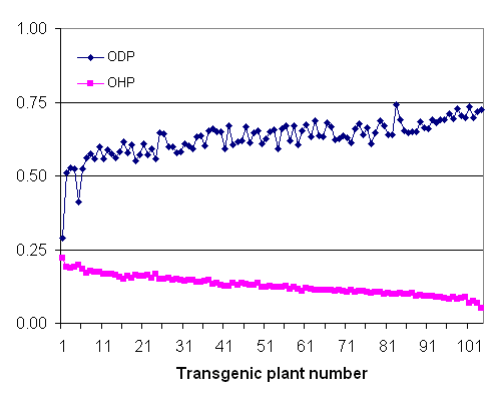
**Comparison of levels of oleate desaturation (ODP) and hydroxylation (OHP) in seeds of 104 *Arabidopsis *transgenic lines co-expressing castor *FAD2 *and *FAH12***. The first plant line is the control, CL37.

The low abundance of *FAD2 *is a surprising contrast with the high level expression of *FAH12*, with 16 ESTs from the total of 4,412 analyzed sequences. This difference in expression level was also confirmed by an RT-PCR analysis (Fig. 5). Since FAD2 and FAH12 act on the same substrate, 18:1-phosphatidylcholine [3], a low level of *FAD2 *expression may favor FAH12 and thus result in a high level of ricinoleate accumulation in castor seeds. To test this idea, we over-expressed the castor *FAD2 *in the CL37 *Arabidopsis *line expressing the *FAH12 *transgene. Indeed, analysis of 104 CL37/FAD2 plant lines demonstrated a negative correlation between levels of desaturation and hydroxylation. As shown in Figure 6, the oleate hydroxylation proportion [OHP = (18:1OH +18:2OH)/(18:1+18:2+18:3+18:1OH+18:2OH)] decreased as the oleate desaturation proportion (ODP = (18:2 +18:3)/(18:1+18:2+18:3 +18:1OH +18:2OH)) increased. The hydroxy fatty acid content (total HFA) is reduced from 17+/-1% in the CL37 parental line to less than 5% in the most-extreme *FAD2 *transgenics (Table 2). This effect is not likely a result of homologous co-suppression since castor *FAD2 *and *FAH12 *are only ~70% identical in nucleotide sequence. This result suggests that castor endosperm is highly specialized to ricinoleate synthesis through the evolution of *FAH12*, a member of the *FAD2 *superfamily [19]. Regulation of *FAD2 *and *FAH12 *expression in castor endosperm may contribute to high-level accumulation of ricinoleate in castor oils. In castor endosperm, expression of *FAD2 *may be kept at minimum to maintain membrane lipid synthesis and normal cell functions. There may be also other *FAD2 *homologs in castor that were not detectable in our EST analyses since we used mRNA from a specific stage of endosperm development. In addition, the FAH12 enzyme has a low level of desaturation activity [20]. Although this scenario may be true in castor endosperm, heterologous expression of *FAH12 *in a FAD2-deficient *Arabidopsis *line (*fad2*) did not result in an increased level of hydroxy fatty acid accumulation in transgenic seeds [20]. Other components in developing castor endosperm probably have co-evolved with the FAH12 enzyme to facilitate hydroxy fatty acid synthesis and assembly into storage oils [6]. The search for such factors is an ongoing process in the authors' laboratories and will benefit from the cDNA library and EST analysis described here.

**Table 2:** Fatty acid compositions of the hydroxylase-transgenic line CL37 and selected lines that were transformed with the additional castor *FAD2 *gene. Data represent mean values of three independent GC analyses

Line	Fatty acid composition (mol%)								ODP	OHP
			
	16:0	18:0	18:1	18:2	18:3	18:1OH	18:2OH	Total HFA		
CL37	13.7	6.3	33.1	22.1	6.3	14.2	3.2	17.4	0.29	0.22
89	11.7	6.0	23.4	35.3	7.4	12.5	3.1	15.5	0.51	0.19
97	11.6	6.1	20.4	38.7	8.3	11.5	2.8	14.3	0.58	0.18
63	11.0	7.2	20.9	39.1	8.3	10.4	2.4	12.8	0.58	0.16
9	10.4	5.9	17.5	44.7	8.6	9.5	2.9	12.4	0.64	0.15
34	10.5	6.0	17.9	44.9	9.2	8.5	2.3	10.8	0.65	0.13
20	10.5	5.3	16.9	47.1	9.6	7.5	2.7	10.2	0.67	0.12
65	10.5	4.8	17.9	46.2	11.1	6.5	2.5	9.0	0.65	0.11
29	9.8	5.3	19.5	47.7	9.9	5.5	1.7	7.3	0.69	0.09
17	10.4	4.4	17.2	49.5	11.6	4.5	1.8	6.3	0.70	0.07
83	12.4	4.0	18.3	48.0	12.2	3.2	0.9	4.1	0.72	0.05

## Conclusion

We report here an analysis of the ESTs derived from a full-length cDNA library of castor developing endosperm. The ESTs are enriched in genes encoding storage proteins, ricin, oleosins, as well as other housekeeping cellular components such as those for protein synthesis. We identified two ESTs of the castor acidic TAG lipases, which are abundantly expressed in developing castor endosperm. Expression of these lipases did not increase ricinoleate accumulation in transgenic *Arabidopsis *seeds. Their function in castor developing seed remains unclear. In contrast to *FAH12*, *FAD2 *is much lower in abundance in our cDNA library, suggesting that regulation of *FAD2 *and *FAH12 *expression in castor endosperm may contribute to high-level accumulation of ricinoleate in castor oils, and our results in transgenic Arabidopsis plants support this possibility.

A full-length cDNA resource is particularly valuable for the correct annotation of genomic sequences and for the functional analysis of genes and their products [6,21,22]. Recently, The Institute for Genomic Research (TIGR) has initiated a project to generate redundant sequence analysis of the castor genome . Our results contribute to a better understanding of the castor plant at the genomic level, most especially for understanding seed metabolism. Future EST work will focus on subtractive or normalized cDNA library material to expedite gene discovery and functional genomic studies. We will also include EST analyses using mRNA extracted from different stages of seed development. Our ultimate goal is to identify genetic factors contributing to increased ricinoleate accumulation in seed oils, first in *Arabidopsis *and ultimately in oilseed crops.

## Methods

### Construction of a full-length cDNA library

A full-length cDNA library was constructed in a lambda vector incorporating the Gateway cloning system [6]. Briefly, developing castor seeds were harvested at 20 days after pollination at developmental stage IV, when the endosperm undergoes rapid dimensional growth and gain in weight [23]. The embryos were removed and total RNA was extracted from the endosperm. After mRNA purification, first strand full-length cDNA was generated with Superscript III reverse transcriptase (Invitrogen) and primer 5'-GAGAGAGAGAGAGAGAGAGGATCCACTCGAG TTTTTTTTTTTTTTTTVN-3' (including the restriction sites for BamHI and XhoI), followed by the cap-trapping procedure described by Carninci and Hayashizaki [24]. Second strand cDNA was synthesized using the Single-Strand Linker Ligation Method [25]. The resulting double-stranded cDNA was digested with SstI and XhoI, then ligated into the digested arms of the λ_GW _cloning vector [6]. The ligation product was packaged with Max Plax (Epicentre, Madison, WI) according to manufacturer's protocol. Consequently, a full-length cDNA library containing ~5 × 10^5 ^clones was obtained.

### Sequencing of a full-length cDNA library

For sequencing, the cDNA library was transferred into the plasmid vector pDONR201 (Invitrogen) by the BP cloning process, then transformed into *E. coli *DH10B by electroporation. With the assistance of the Research Technology Support Facility at Michigan State University, colonies were picked randomly, inoculated into 96-well plates containing 1 mL of LB media and incubated at 37°C for 18 hr. DNA from bacterial cultures was purified using a Qiagen 3000 robot, and cDNA inserts were sequenced once from the 5'end of each clone using the BigDye terminator kit and an automated DNA capillary sequencer (ABI 3730, Applied Biosystems). The sequencing primer (5'-AAAAGCAGGCTGAGCTCGTCG-3') was designed to overlap the cDNA insertion site so that vector sequences were not included in EST sequences.

### Sequence data analysis and EST clustering

The 5' DNA EST sequence chromatogram data were base-called using the program Phred [10]; EST reads were quality trimmed using the Phred quality score at a position where five ambiguous bases (phred quality > 2 and at least 200 bp) were found within 15 consecutive bases. EST sequences were clustered using the software stackPACK (provided by SANBI [26]). Groups that contained only one sequence were classified as singletons. EST sequences longer than 200 bp were compared to NCBI [27] and TAIR [28] databases using the BLASTX program.

### Functional analysis of the *FAD2 *gene

The corresponding open reading frame (ORF) of the castor *FAD2 *gene was amplified by PCR using Phusion DNA polymerase (New England Biolabs) and the following pair of specific primers: 5'-GCAAGCTTATGGGTGCTGGTGGCAGAAT-3' and 5'-GATCTAGATCAAAATTTGTTGTTATACCAG-3'. For ligation behind the inducible GAL1 gene promoter of the yeast expression vector pYES2 (Invitrogen, CA), the primers were extended by a HindIII or a XbaI restriction site (underlined), respectively. The resulting 1.2-kb PCR product was cloned into the vector pYES2 and transformed into the *Saccharomyces cerevisiae *strain DBY747 using the Frozen-EZ Yeast Transformation kit (Zymo Research, CA). Complete minimal drop out-uracil medium containing 2% glucose as the exclusive carbon source was inoculated with a single colony and grown at 30°C over night. FAD2 expression was induced by transferring the cells into the above medium containing 2% galactose instead of glucose, and grown overnight. Yeast cells were harvested by centrifugation at 1500 g for 5 min at 4°C, and washed once with distilled water. Fatty acid analyses were conducted as described below.

For RT-PCR analysis of *FAD2*, 1 μg of mRNA extracted from developing castor endosperm was used to do reverse transcription in 20 μL volume using the SuperScript III first-strand cDNA synthesis system for RT-PCR following the manufacturer's instructions (Invitrogen, CA). PCR was conducted using the above primers specific to castor *FAD2 *gene and 0.5 μL cDNA from the RT reaction. The PCR reaction was initiated by one cycle of 94°C for 3 min, and followed by 15 or 25 cycles of 94°C 30s, 55°C 30s and 72°C 1 min. For amplification of the *FAH12 *gene, the following pair of gene specific primers were used: 5'-ATGGGAGGTGGTGGTCGCAT-3' and 5'-TTAATACTTGTTCCGGTACC-3'. The primers 5'-ATGGCTGAGCATCAACAATCAC-3' and 5'-TCAGCCCTGTCCTTCATCTC-3' were used to amplify the oleosin *OLE2 *gene. All three resulting PCR products are full-length cDNA of the open reading frames.

### Transgenic plant analysis

We have previously described the *Arabidopsis *transgenic line CL37, expressing the castor oleate hydroxylase FAH12 [6]. Full-length cDNA clones of the *RcFAD2 *and lipase genes were cloned into the plant expression vector pGate-DsRed-Phas [6] by the gateway LR cloning process following the manufacturer's instructions (Invitrogen), and transformed into CL37 by an *Agrobacterium*-mediated floral dip method [29]. Transgenic seeds were screened using the DsRed fluorescent protein marker [6,30]. Transgenic red seeds were sorted for comparison to non-transgenic seeds from the same T1 plant, and the fatty acids were analyzed by gas chromatography. Fatty acid methyl esters were prepared by heating ~20 seeds at 80°C in 1 ml 2.5% H_2_SO_4 _(v/v) in methanol for 90 min, followed by extraction with 200 μl hexane and 1.5 ml of 0.9% NaCl (w/v), then 100 μl of the organic phase was transferred to autoinjector vials. Samples of one μl were injected into an Agilent 6890 GC fitted with a 30-M × 0.25-mm DB-23 column (Agilent). The GC was programmed for an initial temperature of 190°C for 2 min followed by an increase of 8°C per min to 230°C and maintained for a further 6 min.

## Authors' contributions

CL and JGW conducted research; CL and JB designed and planned the experiments. All authors were involved in writing the paper, and agreed the final draft.

## Supplementary Material

Additional file 1 BLAST results of unique castor cDNA sequences. BLAST results of 1,908 unique sequences from a full-length cDNA library of developing castor endosperm. Click here for file
